# Deregulation of MiR-34b/Sox2 Predicts Prostate Cancer Progression

**DOI:** 10.1371/journal.pone.0130060

**Published:** 2015-06-24

**Authors:** Irene Forno, Stefano Ferrero, Maria Veronica Russo, Giacomo Gazzano, Sara Giangiobbe, Emanuele Montanari, Alberto Del Nero, Bernardo Rocco, Giancarlo Albo, Lucia R. Languino, Dario C. Altieri, Valentina Vaira, Silvano Bosari

**Affiliations:** 1 Division of Pathology, Fondazione IRCCS Ca' Granda—Ospedale Maggiore Policlinico, Milan, Italy; 2 Department of Pathophysiology and Organ Transplant, University of Milan, Milan, Italy; 3 Department of Biomedical, Surgical and Dental Sciences, University of Milan, Milan, Italy; 4 Department of Health Science, University of Milan, Milan, Italy; 5 First Division of Urology, San Paolo Hospital, Milan, Italy; 6 Division of Urology, Fondazione IRCCS Ca' Granda—Ospedale Maggiore Policlinico, Milan, Italy; 7 Prostate Cancer Discover and Development Program, The Wistar Institute, Philadelphia, PA, United States of America; 8 Department of Cancer Biology, Kimmel Cancer Center, Thomas Jefferson University, Philadelphia, PA, United States of America; 9 Tumor Microenvironment and Metastasis Program, The Wistar Institute, Philadelphia, PA, United States of America; 10 Istituto Nazionale di Genetica Molecolare 'Romeo ed Enrica Invernizzi', Milan, Italy; Innsbruck Medical University, AUSTRIA

## Abstract

Most men diagnosed with prostate cancer will have an indolent and curable disease, whereas approximately 15% of these patients will rapidly progress to a castrate-resistant and metastatic stage with high morbidity and mortality. Therefore, the identification of molecular signature(s) that detect men at risk of progressing disease remains a pressing and still unmet need for these patients. Here, we used an integrated discovery platform combining prostate cancer cell lines, a Transgenic Adenocarcinoma of the Mouse Prostate (TRAMP) model and clinically-annotated human tissue samples to identify loss of expression of microRNA-34b as consistently associated with prostate cancer relapse. Mechanistically, this was associated with epigenetics silencing of the MIR34B/C locus and increased DNA copy number loss, selectively in androgen-dependent prostate cancer. In turn, loss of miR-34b resulted in downstream deregulation and overexpression of the “stemness” marker, Sox2. These findings identify loss of miR-34b as a robust biomarker for prostate cancer progression in androgen-sensitive tumors, and anticipate a potential role of progenitor/stem cell signaling in this stage of disease.

## Introduction

Prostate cancer (PCa) is the most commonly diagnosed visceral malignancy among males worldwide. In the United States, PCa accounts for 26% of total new cancer cases and 9% of the total cancer-related deaths, ranking second to lung cancer. The probability of developing prostate cancer from birth to death is 1 in 7, with the highest incidence in men over 70 years of age [1]. Although most men diagnosed with prostate cancer will have an indolent and curable clinical course, approximately 15% of these patients will display a rapidly progressing, treatment-resistant and metastatic disease to bones and visceral organs with high morbidity and mortality [2]. As no reliable means of identifying patients at risk of metastatic dissemination are currently available [3], the search for molecular signature(s) of disease progression, especially transition to castrate-resistance [4] is an urgent and still largely unmet medical need in managing a prostate cancer diagnosis.

MicroRNAs (miRNAs) are small, non-coding, endogenous RNAs with pleiotropic functions in gene expression [5,6]. This pathway is dramatically exploited in cancer, and a variety of miRNAs have been linked to deregulated oncogenic or tumor suppressor pathways [7,8]. Reciprocally, drivers of malignancy, including DNA aberrations, transcriptional deregulation and epigenetics silencing contribute to aberrant miRNA expression [9,10]. For their stability in biological fluids [11], and relative ease of detection in clinical samples [12], miRNAs have been pursued as tumor biomarkers [8], for their potential predictive or prognostic value in patients with breast [13], liver [14,15], or lung [16] cancer. Deregulated miRNA expression has also been observed in prostate cancer, compared to normal epithelium [17–19], and in some cases linked to metastatic dissemination [20–22], but definitive signature(s) of disease progression, especially castrate-resistance, have not been identified.

In this study, we used an integrated discovery platform combining established cell lines, a genetic mouse model of disease progression, and primary patient samples to profile differential miRNA expression in prostate cancer.

## Materials and Methods

### Clinical specimens

The clinical and pathological parameters of PCa patients or benign prostatic hyperplasia (BPH) examined in these studies are described in Table 1. Globally, we retrieved archival tissues and clinical records from 192 patients who underwent surgery from 2004 to 2006 at San Paolo Hospital or Fondazione IRCCS Ca’ Granda Hospitals in Milan (Italy), under a protocol approved by the Institutional Review Boards (IRB) of San Paolo Hospital (code 10664) and Fondazione IRCCS Ca’ Granda-Ospedale Maggiore Policlinico (code 1381/11). Because of the retrospective nature of this study and the use of data anonymization practices, the need for written informed consent was waived. Follow-up consisted in active surveillance of the patients by consecutive measurement of serum prostatic-specific antigen (PSA) levels. For PCa patients, biochemical recurrence was defined as two consecutive PSA levels greater than 0.2 ng/ml.

**Table 1:** Clinical characteristics of prostate cancer patients used in the indicated analysis.

	Patients’ Series			
Characteristic	Series A (n = 50)	Series B (n = 16)	Series C (n = 192)	Series BPH (n = 10)
*Age*	* *	* *	* *	* *
Mean±SD	64±7.1	65±6.4	65.5±6.3	69±5.5
Range	50–75	53–74	44–77	57–77
*Gleason score *	* *	* *	* *	* *
<7	19	2	62	-
7	24	6	97	-
>7	7	7	29	-
*Na*	-	1	4	-
*T-stage*	* *	* *	* *	* *
T2	35	5	124	-
T3-T4	15	11	68	-
*Lymph node status *		* *	* *	* *
N0	47	5	167	-
N1	2	8	16	-
Nx	1	3	9	-
*Perineural invasion*		* *	* *	* *
Yes	36	11	112	-
No	13	5	56	-
*Na*	1	-	24	-
*Biochemical recurrence*		* *	* *	* *
Yes	6	6	26	-
No	44	4	107	-
*Na*	-	6	59	-
*PIN lesion*		* *	* *	* *
	50	0	50	-

*Na*: *not available*.

For molecular analyses cases must had satisfactory RNA contents (260/280 Absorbance Ratio >1.2 and <2.1 and 500 ng of total RNA) in at least a matched normal and tumor prostate sample per patient. Sixty-six patients out of the 192 assessed satisfied these criteria and for fifty of those also prostatic intraepithelial neoplasia (PIN) lesion was available. Therefore we sorted patients in a first subset with complete availability of normal, PIN and PCa tissues (n = 50, series A; Table 1), and in a second subset composed of the remaining patients with only matched normal and tumor samples (n = 16, series B; Table 1). Epithelial tissues for normal prostate, PIN and PCa were separately retrieved by punching archival blocks with a 1 mm diameter needle as described [23]. Each sample consistently exceeded 80% purity in epithelial cell content. Normal tissue sampling was performed at a distance of at least 2 cm from neoplastic tissue. The full set of patients enrolled in this study (n = 192, series C; Table 1) was used to build sixteen tissue micro arrays (TMA) blocks [24] for immunohistochemical evaluations.

BPH samples (n = 10) were patients who underwent transurethral resection of prostate (TURP) at Fondazione IRCSS Ca’ Granda Hospital (Milan) for whom prostatic mapping was negative for PCa and followed up for at least 3 years (series BPH; Table 1). This series was not included in the TMA platform and full tissue sections were used for either molecular or immunohistochemical analyses.

### Cell lines

Human prostate cell lines RWPE-1, BPH-1, LNCaP, DU145 and PC3 cells were obtained from the American Type Culture Collection (ATCC, Manassas, VA). The normal cell line RWPE-1 was cultured in Keratinocyte Serum-Free Medium supplemented with 5 ng/ml EGF, 0.05 mg/ml bovine pituitary extract, and 1% Pen-Strep antibiotics cocktail. All other cell lines were cultured in RPMI supplemented with 10% FBS and 1% Pen-Strep. Cell cultures were maintained at 37°C in a 5% CO_2_ incubator. For miRNA transfection, cells were seeded at a 5x10^5^ per well in six-well plates, and transfected with 150 pmol of miR-34b inhibitor (a-miR-34b; HSTUD0511), or miR-34b precursor (p-miR34b; HMI0511), or corresponding non-targeting sequences (a-Ctrl or p-Ctrl, respectively HMC0002 and NCSTUD001) in the presence of Lipofectamine 2000 (Life Technologies Inc., Carlsbad, CA, USA), as described [25]. All miRNA molecules were from Sigma Aldrich, Milan, Italy.

### Laser-assisted microdissection of prostate lesions from Transgenic Adenocarcinoma of the Mouse Prostate (TRAMP) mice

Archival tissue blocks of the urogenital tract, including bladder, seminal vesicles and prostate from five TRAMP mice were used [26,27]. All mice had developed prostatic tumors and were sacrificed at 24 weeks of age. All animal experiments have been reviewed and approved by an Institutional Animal Care and Use Committee at Thomas Jefferson University. Discomfort and injury to animals have been limited to that which is unavoidable in the conduct of scientifically valuable research. All experimental procedures carried out as part of this study comply with approved protocols to ensure the highest standards in humane care to the animals. Analgesics, anesthetics and tranquilizing drugs have been used as determined by the veterinary staff. Euthanasia has been performed out by CO_2_ anesthesia. This method is consistent with the recommendations of the 1316 Panel of Euthanasia of the American Veterinary Medical Association.

Prostate samples from TRAMP mice were enriched in PIN or prostatic tumors (S1 Fig) by laser-assisted microdissection (LMD; Leica Microsystems, Milan, Italy) of epithelial lesions, as described [28]. For miRNA profiling, PCa or PIN tissues from different animals (n = 5 per group) were pooled.

### RNA purification, retrotranscription and microRNA profiling

Total RNA was isolated using the MasterPure RNA Purification Kit (Epicentre Biotechnologies, Madison, WI, USA), as described [28], and retrotranscribed using the Megaplex RT primers Human or Rodent pools A and B v3.0 and Taqman MicroRNA Reverse Transcription kit. Murine samples were pre-amplified using Megaplex PreaAmp primer Rodent pools A and B. Human or rodent TaqMan low-density arrays (TLDA) were then performed for miRNA profiling in prostate cancer cell lines or TRAMP prostatic tissues, respectively. Human TLDA includes 754 miRNAs and 4 endogenous nucleolar RNAs (RNU44, RNU48 and U6snRNA), whereas Rodent TLDA includes 750 miRNAs, and 6 control RNAs, of which 675 miRNAs and three endogenous controls (snoRNA135, snoRNA202 and U6snRNA) are specific for mouse. Both Human and Rodent platform includes one negative control probe (ath-miR-159a) per card. For validation, eighteen selected miRNAs plus reference transcripts (U6snRNA, RNU48, RNU44, and RNU24) and two negative controls (ath-miR-159a and a well without detection probe) were analyzed in human samples (series A, B and BPH; Table 1) using a custom RT and PreAmp primers pools together with a custom TLDA platform with pre-spotted primers and Taqman probes. All reagents, kits and instrumentation used for miRNA profiling were from Life Technologies Inc. (now part of Thermo Scientific, Carlsbad, CA, USA).

### Methylation analysis of MIR34B/C CpG island

Genomic DNA was purified from 4 prostate cell lines (RWPE-1, BPH-1, LNCaP, DU145), 16 PCa, and 12 normal prostate tissues (from series B), and from 10 BPH (series BPH) samples using the QIAamp DNA Mini Kit (Qiagen, Waltham, MA, USA). Sodium bisulphite conversion of DNA (400 ng) was performed using the EZ DNA Methylation-Gold Kit (Zymo Research Corporation, Irvine, CA). Methylation-specific polymerase chain reaction (MSP) of a CpG island upstream the MIR34B/C locus was performed as described [29], using the following primers for detection of unmethylated (UM) or methylated (M) region: UM-forward 5’- TGTTTTTTGGTGAAATGGGGTTTGAGGT-3’ UM-reverse 5’- CCTACAAACCAAACACCAAACACCCACA3’; M-forward 5’- CGGTGAAATGGGGTTCGAGGC-3’ M-reverse 5’-CCGAACACCGAACACCCGCG-3’. The thermal profile was 95° for 10 min followed by 95°C for 1 min, 65°C for 1 min, and 72°C for 1 min for 45 cycles and then 72°C for 7 min.

### Copy number variation (CNV) analysis

Genomic variations of 11q23.1b cluster and adjacent loci were analyzed by TaqMan copy number assay relative to the reference gene RNase P, as described [30]. The assay identification numbers were as follows: Hs00608392_cn (CNV#1; exact location 111383042), Hs03049129_cn (CNV#2; exact location 111368721) and Hs06336326_cn (CNV#3; exact location 81902545). Variations in DNA copy number were quantified using Copy Caller software. All assays, reagents and software were from Life Technologies Inc.

### miR-34b targets expression analysis

Hyperplastic (BPH-1) or tumor (LNCaP and DU145) prostate cell lines were transfected with miR-34b mimic/inhibitor or controls for 72 hours and total RNA was purified as described above. Then, DNA-free total RNA was reverse transcribed with random hexamers and SOX2, CDKN1A, MET, c-MYC, HES1 genes expression (TaqMan Gene Expression Assays) was relatively quantified on a reference transcript (beta-2-microglobulin) by Real-Time PCR (qPCR). All reagents and instruments were from Life Technologies Inc. Target expression was then calculated using the 2^^-ΔCt^ formula, median normalized and log2 transformed for heat map generation using dChip softare (DNA-Chip Analyzer, www.dchip.org) as described [9].

### Immunoblotting

Cells were harvested 72 h after transfection of miRNA mimics/inhibitor and solubilized in 100 μl lysis buffer supplemented with protease inhibitor cocktail (Roche, Basel, Switzerland), as described [25]. Aliquots (50 μg) of each cell lysate were probed with 1 μg/ml of the following primary antibodies to c-Myc (clone D84C12; Cell Signaling Technologies, Danvers, MA, USA), Sox2 (clone D6D9; Cell Signaling Technologies), or Notch1 (clone A-8; Santa Cruz Biotechnologies, Santa Cruz, CA, USA). β-tubulin (Sigma Aldrich) was used as a loading control. Reactive bands were visualized with ECL Plus (GE Healthcare, Milan, Italy).

### Prostate Tissues Microarrays (TMA) construction and Immunohistochemistry (IHC)

From each of the 192 PCa patients (series C; Table 1), four cores of prostate tumor, one core of PIN and one core of normal parenchyma if available were used to build 22 TMA blocks, as described [24], with modifications [23]. FFPE tissues from patients with BPH (series BPH; Table 1) were used as full sections and were not included in the TMA. Four μm-thick sections from each block were stained with an antibody to Sox2 (1:100; Cell Signaling), with diaminobenzidine (DAB) as a chromogen. Immunohistochemistry was performed using Benchmark Ultra Roche Ventana immunostainer (Roche Group, Tucson, AZ, USA). All slides were counterstained with hematoxylin. Two pathologists (GG and SF) blinded to clinical data evaluated and scored all slides. When discrepancies occurred, the case was further reviewed to reach an agreement score. Only reactivity for nuclear Sox2 in epithelial cells and not in basal myoepithelial compartment was recorded and the percentage of immunoreactive cells in PCa samples was averaged among the four cores from the same patient. In line with previous report [31], Sox2 immunoreactivity was then categorized in four groups and scores were then assigned as follows: score 0 (negative nuclear staining), 1 (1–10% of nuclear Sox2), 2 (11–50% of nuclear Sox2), 3 (more than 50% of nuclear Sox2).

### Statistical Analysis

MiRNAs relative quantities (RQ) were obtained importing raw TLDA data files in DataAssist software (Life Technologies Inc.) using a value of 35 as threshold for maximum allowable Ct and global normalization for target quantification. RQ values were then log2 transformed and imported in BRB ArrayTools (http://linus.nci.nih.gov/BRB-ArrayTools.html) where Anova or differential expression analyses were performed, as described [15]. Correlation parameters between miRNA expression and clinicopathological variables were derived using Mann-Whitney U test, Friedman test or chi-square test for continuous or discrete variable, respectively, using GraphPad Prism (GraphPad Software, Inc., La Jolla, CA) or MedCalc (Mariakerke, Belgium) statistical package. Receiver operating characteristics (ROC) curves method was used to test the accuracy of miRNAs to correctly discriminate between benign disease, PIN or prostate cancer, and to identify patients who had PSA failure (PSA> 0.2 ng/ml for at least two consecutive times) during follow-up, as described [15]. dChip software was used for unsupervised hierarchical clustering. *In vitro* experiments were performed at least three times and data are expressed as the mean±SD unless otherwise specified. A p value <0.05 was considered as statistically significant.

## Results

### miRNA signatures of Prostate cancer models

**Fig 1:**
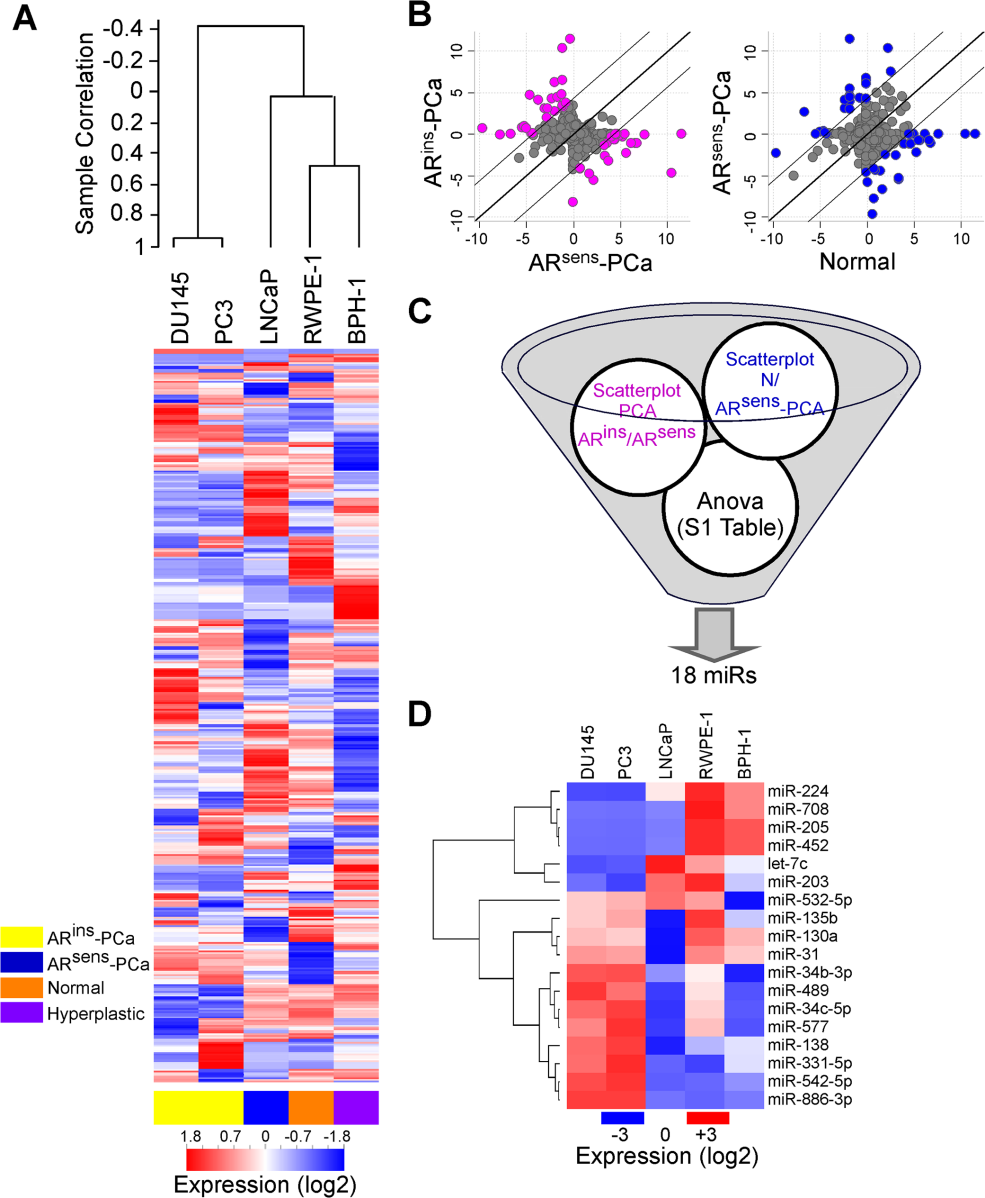
miRNA expression in prostate cell lines. **A**) Heat-map of global miRNA profiles (≈750 miRNAs) in non-tumorigenic (RWPE-1, Normal), hyperplastic (BPH-1), androgen–dependent (LNCaP, AR^sens^-PCa) or–independent (DU145 and PC3, AR^ins^-PCa) prostate cancer cells. Red, over-expression; blue, under-expression, respectively. **B**) Scatterplots diagrams were performed between androgen–dependent and–independent PCa cells and between normal prostate cells and AR^sens^ PCa cells to select miRNAs with differential expression in either direction (fold-change) of at least 20 folds. A list of 41 miRNAs simultaneously altered in both comparisons was generated. **C)** Schematic diagram of selection criteria adopted to identify miRNAs potentially related to PCa. **D**) The eighteen miRNA signatures identified in **C** were verified by qPCR in the indicated prostate cells. All miRNAs except two (miR-577 and miR-886-3p) had mouse orthologs. Red, white and blue colors in the heat-map represent higher, equal or lower miRNA expression in samples respect to median miRNA value.

We began this study by profiling the expression of miRNA in a panel of prostate cancer cell types with different tumorigenic potential (S1 File) and TRAMP mice (S2 File). In these experiments, miRNAs expression readily differentiated androgen-insensitive (DU145 and PC3) from-sensitive prostate cancer cell types (Fig 1A). Accordingly, non-tumorigenic RWPE-1 and BPH-1 cell lines (Fig 1A) clustered separately from more aggressive, LNCaP cells. We next selected miRNAs based on two criteria: a 20-fold difference between androgen-sensitive or–insensitive cell types and non-tumorigenic RWPE-1 or tumorigenic LNCaP (Fig 1B) (i), and a significant variation (p<0.05 by Anova) in expression between normal, hyperplastic, androgen–sensitive and–insensitive cells (Fig 1C) (ii). These analyses identified 18 miRNAs, and 16 of these transcripts had mouse orthologs (Fig 1D).

**Fig 2:**
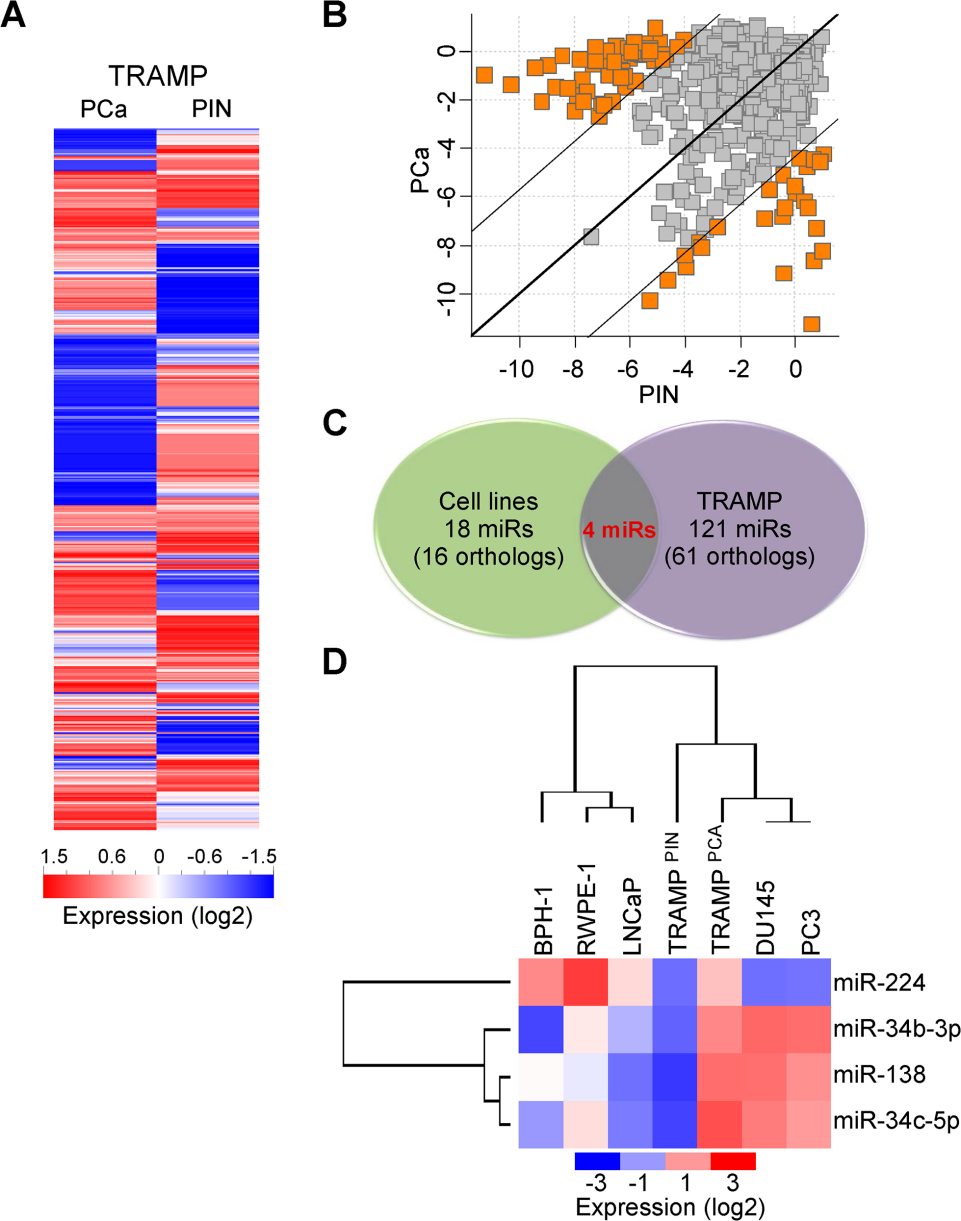
miRNA expression in TRAMP-derived tissues. **A)** Global miRNA profiling of prostate cancer (PCa) or prostatic intraepithelial neoplasia (PIN) lesions isolated from TRAMP mice. **B**) Scatterplot diagram of miRNAs with differential expression of at least 20 folds between PIN and PCa tissues of TRAMP mice. Of the identified 121 deregulated miRNAs (S1 Table) 61 had human orthologs. **C, D**) Comparison of significant miRNA signatures between prostate cell lines and TRAMP lesions. Only four miRNAs were in common between the two experimental systems. Expression analysis followed by unsupervised hierarchical clustering **D**) of miR-224, -34b-3p, -138 and miR-34c-5p reveals that androgen-independent prostate cancer cells are more similar to TRAMP tissues than to androgen-dependent or non-tumorigenic prostate human cells.

We next performed global miRNA profiling of LMD PIN or tumor lesions from TRAMP mice (Fig 2A), using a 20-fold cutoff difference in miRNA expression between the two samples (Fig 2B). One hundreds twenty-one miRNAs fulfilled these criteria (Fig 2B and 2C), and 61 miRNAs had human orthologs (S1 Table). In this analysis, only four miRNAs were differentially expressed in both human prostate cancer cell lines and tumor samples from TRAMP mice, including miR-34b-3p, miR-34c-5p, miR-138, and miR-224 (Fig 2C and 2D). Accordingly, the expression profile of these four microRNAs demonstrated that androgen-independent DU145 or PC3 cells were more closely related to TRAMP tumors, than other human cell lines (Fig 2D).

### LNCaP PCa model mirrors miRNA deregulation in human prostatic disease

**Fig 3:**
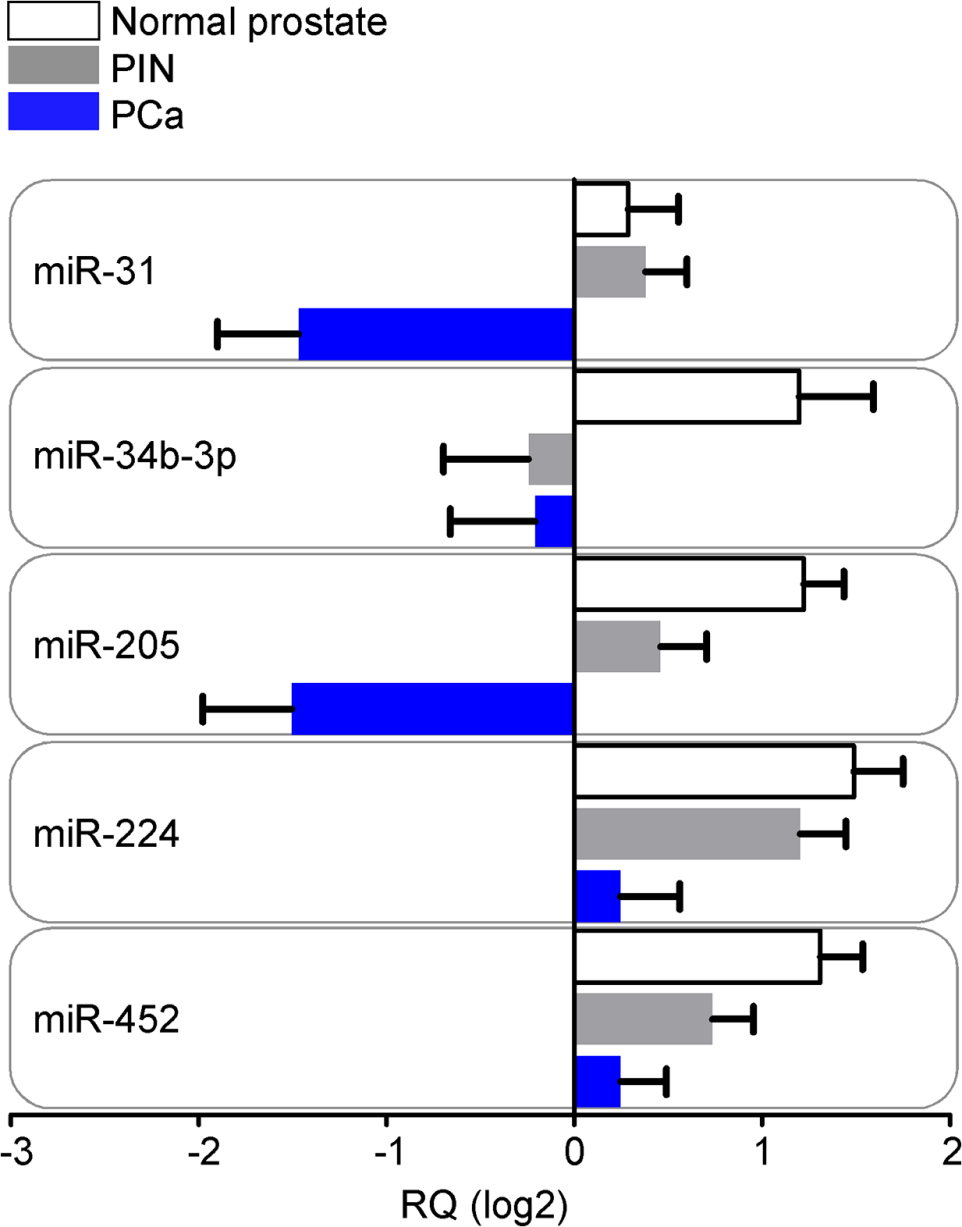
miRNA expression in human prostate tissues. Matched normal prostatic glands, prostatic intraepithelial neoplasia (PIN) and prostate cancer (PCa) lesions available from 50 patients were tested for expression levels of the eighteen selected miRNAs. MiR-31, 34b-3p, 205, 224 and miR-452 displayed significantly altered levels between the three tissue classes (p<0.01 by Friedman test). Bars, mean±SEM. RQ, relative miRNA quantity.

We next examined the miRNAs identified above (n = 18) in a series of 50 patients (series A; Table 1) with a spectrum of lesions including PCa, PIN and normal epithelium. MiR-31, miR-34b-3p, miR-205, miR-224 and miR-452 showed differential expression levels between normal, PIN and PCa matched samples (p<0.01 by Friedman test; Fig 3). All miRNAs were down-regulated in PCa compared to normal epithelium, matching the results obtained with LNCaP, RWPE-1 or BPH-1 cell types (Fig 1D).

**Fig 4:**
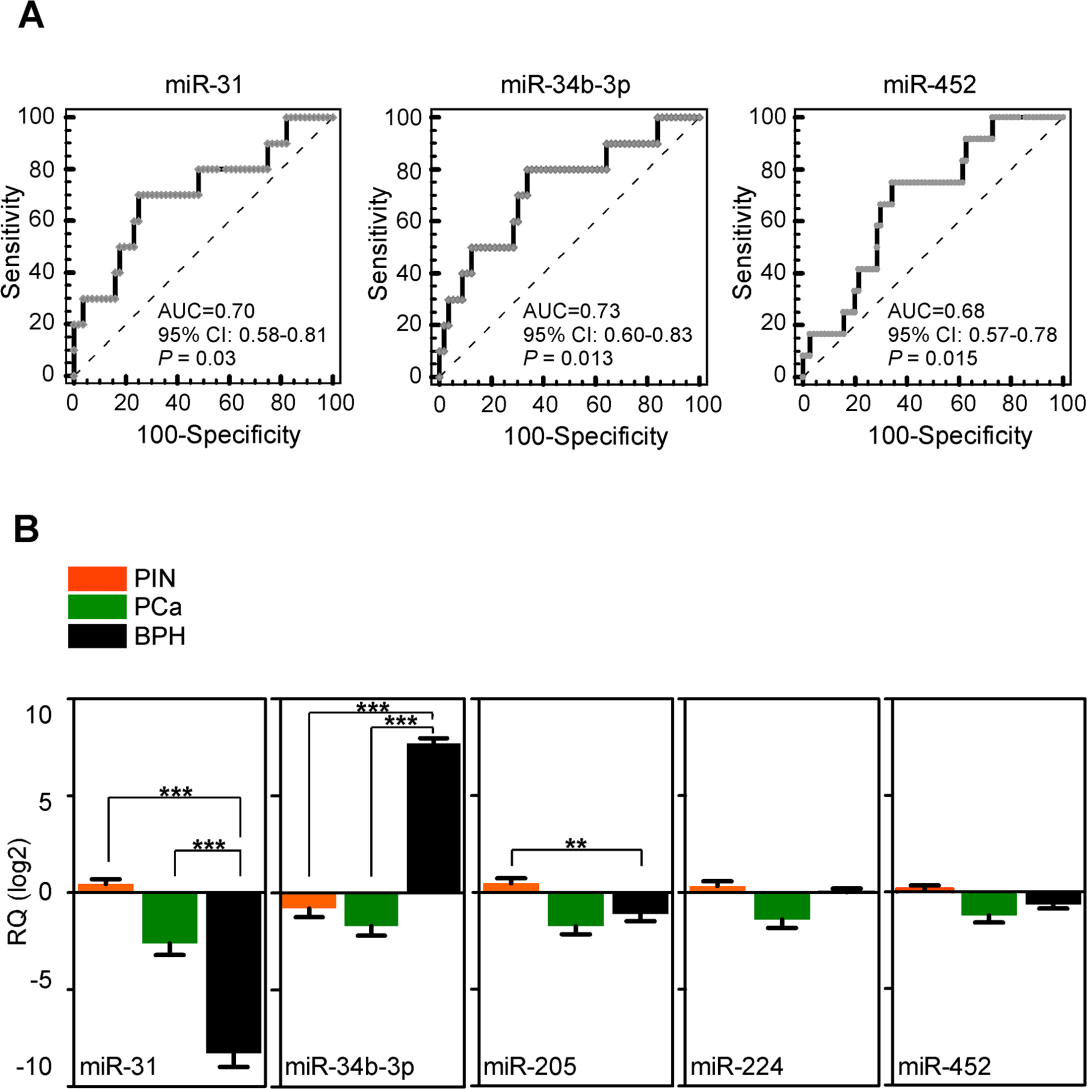
miR-34b-3p is a biomarker of prostate cancer progression. **A)** Receiver operating characteristic (ROC) curves were used to test the accuracy of miRNAs to identify patients who had PSA failure during follow-up. AUC; Area under the curve; CI, Confidence interval. **B**) MiR-31, 34b-3p, 205, 224 and miR-452 expression was evaluated by qPCR in unpaired pre-cancerous glands (PIN, n = 50), neoplastic prostate lesions (PCa, n = 66), or benign hyperplastic tissues (BPH, n = 10). **, p = 0.0027; ***, p<0.001, all by Mann-Whitney test. Bars, mean±SEM. RQ, relative miRNA quantity.

We next focused on these five miRNAs, and examined their potential association with clinical parameters of PCa progression, including biochemical recurrence (two consecutive PSA values >0.2 ng/ml during follow-up), Gleason score (GS ≤7 *vs* GS >7), tumor size (T2 *vs* T3-T4 PCa) and lymph nodes metastases. For further validation, we used an additional, independent set of sixteen patients (series B in Table 1) for an overall number of 66 analyzed PCa. In this analysis, reduced miRNA levels correlated with clinico-pathological progression of PCa (Table 2), and differential expression of mir-31, miR-34b-3p and miR-452 could significantly discriminate patients according to biochemical relapse (Fig 4A). Next, we asked whether the same set of five miRNAs could discriminate between PIN and PCa lesions, thus similar to the relationship between BPH-1 and LNCaP cells. For these experiments, we evaluated ten patients who underwent TURP for BPH (series BPH in Table 1), and were followed up for at least 3 years at Fondazione IRCCS Ca’ Granda Hospital. Both miR-31 and miR-34b-3p were differentially expressed between PIN or PCa samples and BPH (p<0.001 by Mann Whitney test; Fig 4B). Differently from the profile observed in cell lines, miR-34b-3p levels increased more than ten-folds in BPH samples compared to PIN or PCa. Lastly, expression of miR-34b-3p accurately discriminated PIN or PCA samples from BPH, by ROC analysis (p<0.0001; S2 Fig). MiR-205 expression level was lower in BPH compared to PIN (p = 0.0027; Fig 4B). Conversely, miR-452 and miR-224 were not differentially expressed between PCa and BPH or between PIN and BPH, respectively (Fig 4B).

**Table 2:** Correlation of miRNA expression to clinical variables of prostate cancer patients.

			Clinical variable				
miRNA	miRBase ID(v21)	Chr. location	Gleason score(GS ≤7 vs GS >7)	Tumor size(T2 vs ≥T3)	Lymphnodal status(N0 vs N1)	Perineural invasion	Biochemical recurrence
miR-31	MIMAT0000089	9p21.3	0.09	0.074	0.0004	0.34	0.037
miR-34b-3p	MIMAT0004676	11q23.1	0.024	0.065	< 0.0001	0.82	0.021
miR-205	MIMAT0000266	1q32.2	0.44	0.12	0.0064	0.14	0.21
miR-224	MIMAT0000281	Xq28	0.01	< 0.0001	< 0.0001	0.046	0.09
miR-452	MIMAT0001635	Xq28	0.029	< 0.0001	< 0.0001	0.32	0.043

The first and second set of patients (series A and B, n = 66) were grouped. *P* values are from Mann-Whitney U test.

### MiR-34b repression is a biomarker of PCa

**Fig 5:**
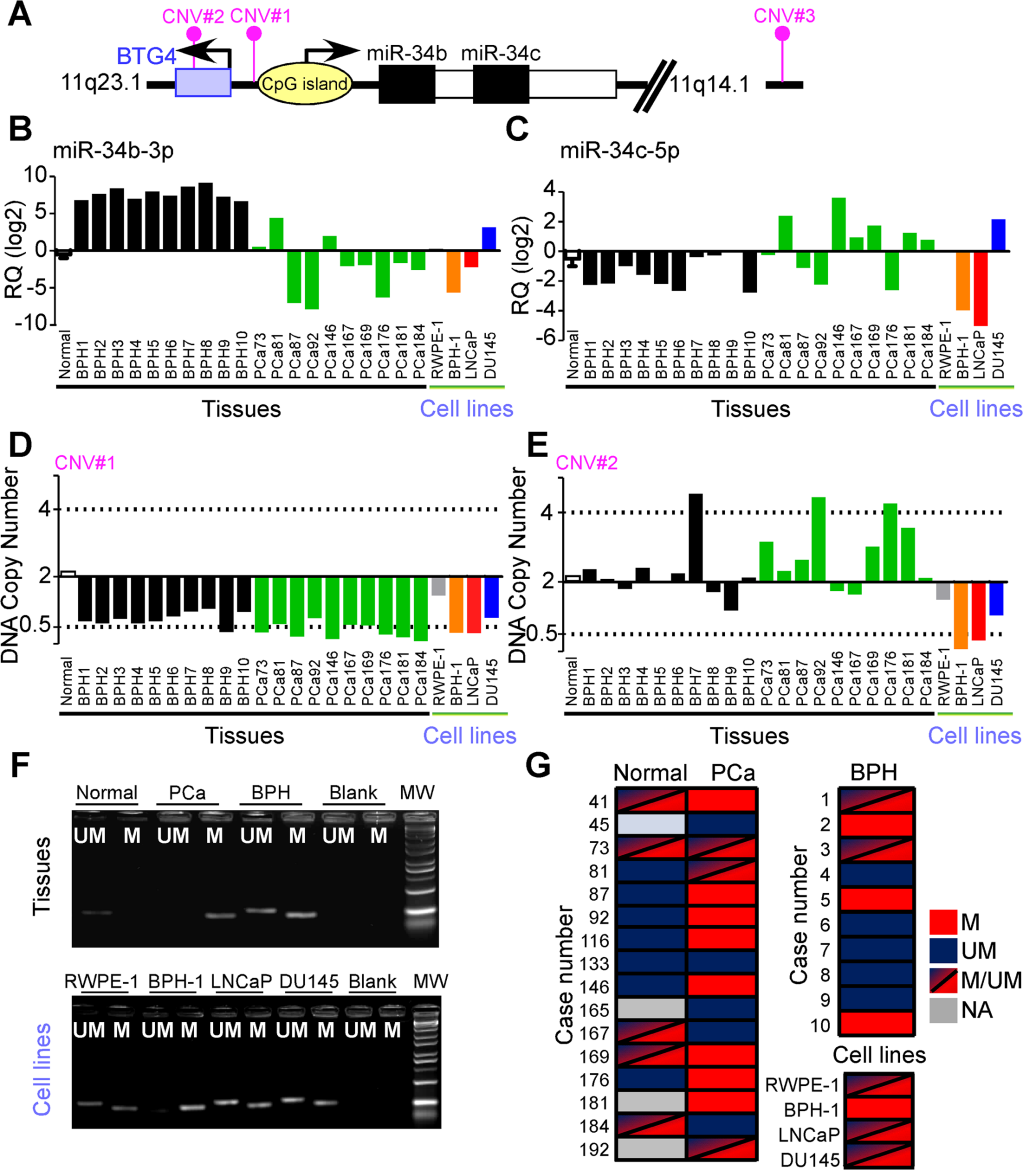
MIR34B/C gene analysis in prostate cells and human tissues. **A)** Schematic diagram of human MIR34B/C locus on chromosome 11q. **B, C)** miR-34b-3p and miR-34c-5p expression in the indicated samples of normal prostate (pool of 10 specimens), benign hyperplasia (BPH), prostate cancer (PCa) or non-neoplastic (RWPE-1), hyperplastic (BPH-1) or tumor (LNCaP, DU145) prostate cell lines. **D, E**) Copy number of genomic DNA regions proximal to MIR34B/C locus (CNV#1; Hs00608392), or to the BTG4 gene (CNV#2, Hs03049129) was evaluated by qPCR in the same samples described in panels **B, C**. Cutoff values for significant loss or gain of targets DNA relative to a reference assay (RNase P) were defined as copies <0.5 or >4, respectively (no change: CNV = 2). **F, G**) Analysis of MIR34B/C CpG island epigenetic status was performed by methylation specific PCR in matched normal or neoplastic (PCa) prostate parenchyma, benign hyperplastic samples and prostate cell lines. A sample was considered partially methylated (M/UM) if amplified by both primer pairs. M, Methylated; UM, Unmethylated. Blank, no template control; NA, sample not available.

MiR-34b and miR-34c are transcribed from the same locus on chromosome 11 (cytogenetic band 11q23.1; Fig 5A), and their expression is regulated by epigenetics, such as CpG island methylation [29], and p53 function [32]. Therefore, we examined the BPH patients, a subset of PCa samples (randomly selected from series B; Table 1), normal prostate specimens, and non-tumorigenic or invasive prostate cell lines for potential allelic copy number variation, and methylation status of the CpG island of the MIR34B/C locus (Fig 5B–5G).

In these experiments, miR-34b-3p was decreased in PCa, compared to normal or hyperplastic prostate samples, BPH-1 or LNCaP cells (Fig 5B). In contrast, miR-34c-5p had a different pattern of expression in human tissues, suggesting that the two transcripts may be independently regulated (Fig 5C). Genomic analysis revealed that only the region proximal to the MIR34B/C locus (assay CNV#1; Fig 5D) exhibited loss of DNA copy number, as the more distant probes (CNV#2 and CNV#3) showed no changes in genomic content (Fig 5E and S2 Table). Loss of DNA copy number was significantly more pronounced in PCa tissues, compared to benign hyperplastic glands (p = 0.01 by Fisher exact test), and was undetectable in the pool of normal controls (Fig 5D). Both CNV#1 and #2 were lost in BPH-1 and LNCaP cell lines, potentially reflecting simultaneous down-regulation of miR-34b/c. In contrast, DU145 cells did not exhibit modulation of miR-34b/c expression, or allelic copy number loss (Fig 5D and 5E).

When analyzed for epigenetic status by methylation-specific PCR, the CpG island upstream of the MIR34B/C locus (Fig 5F) was significantly more methylated in prostate cancers than normal or hyperplastic prostate samples (50% versus 30% or 0% respectively, p = 0.026 by chi-square test; Fig 5G). A partial degree of methylation could be detected in all cell lines but BPH-1, in which MIR34B/C locus was epigenetically silenced (Fig 5F and 5G). Together, these data suggest that decreased expression of miR-34b in PCa may involve a combination of epigenetic silencing and DNA copy number loss (S2 Table).

### A miR-34b-3p-SOX2 axis is selectively deregulated in androgen-dependent PCa

**Fig 6:**
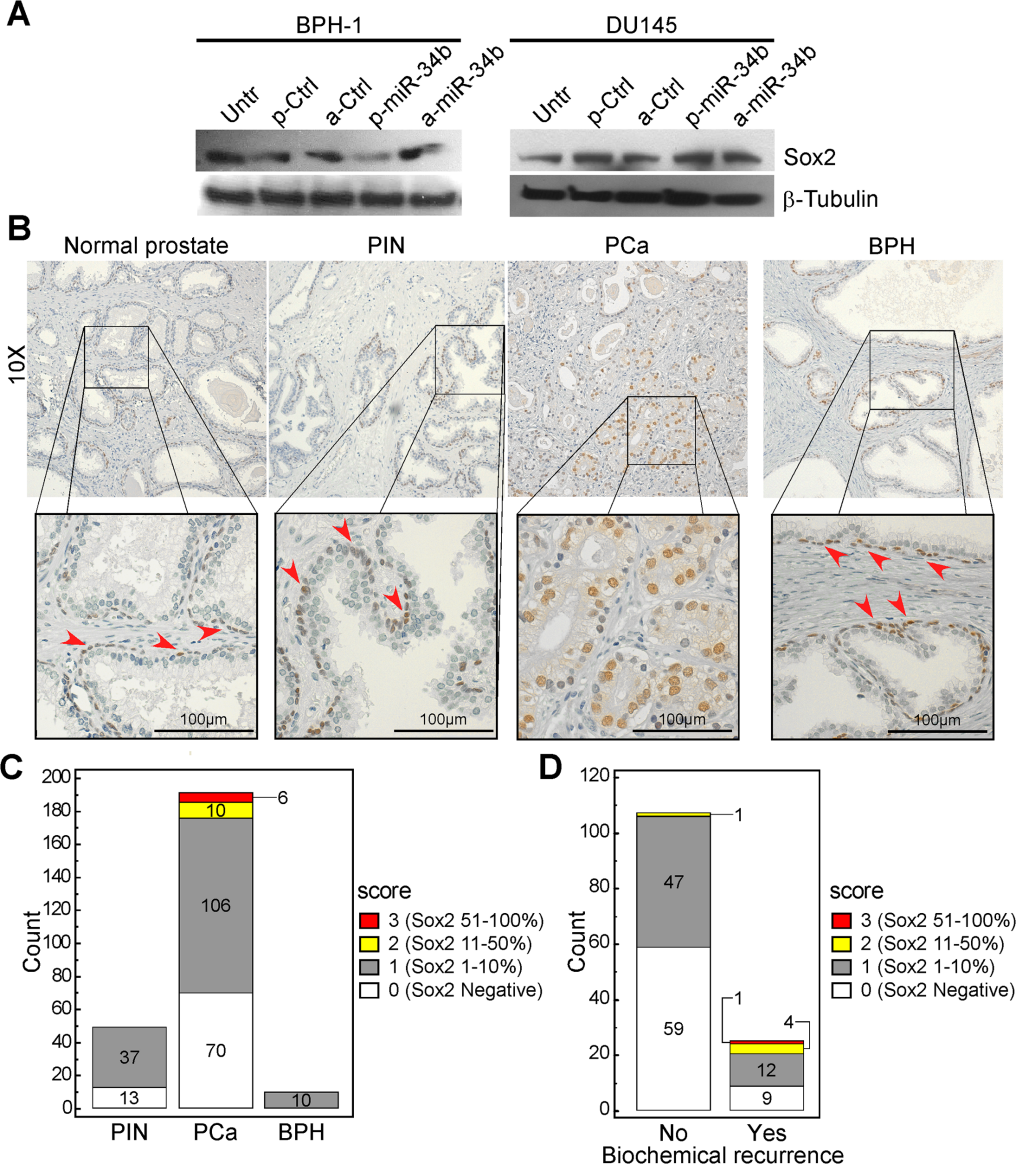
Sox2 is a miR-34b target in prostate cancer. **A)** Sox2 expression was analyzed by immunoblotting in BPH-1 or DU145 cells after transfection with miR-34b mimics or control sequence for 72 h. Untr., untreated sample. p-miR-34b or a-miR-34b, precursor or antagonist miR-34b; p-Ctrl or a-Ctrl, non-targeting controls for precursor or antagonist molecules. **B**) Sox2 immunoreactivity was analyzed in a prostate TMAs, which included normal tissues, pre-neoplastic (PIN) and tumor (PCa) lesions from 192 patients, and in benign prostatic hyperplasia (BPH) samples. Sox2 is highly expressed in basal cell layer from non-neoplastic tissues and in epithelial cells in cancer tissue. **C, D**) Sox2 elevated expression characterizes PCa tissues from PIN or BPH lesions and distinguishes PCa patients with biochemical relapse. The number of PIN, PCa or BPH cases (C) or of PCa patients who underwent or not biochemical relapse during clinical follow-up (serological PSA>0.2; D) according to nuclear Sox2 staining scores is illustrated. p = 0.02 or p = 0.0006, respectively by Chi-square test.

MiR-34b-3p regulates the expression of stem cell-related factors, including c-Myc, Sox2, Met and Notch1 [33,34], which have also been implicated in prostate cancer [35–37]. Consistent with these observations, forced expression of miR-34b precursor sequences in different prostate cell lines but not antagonist (Panel A in S3 Fig), potently repressed endogenous Sox2 levels in BPH-1 cells (Fig 6A) whereas did not modulate the expression levels of c-Myc, Notch1 and Met (Panels B,C in S3 Fig). Conversely, androgen-independent DU145 cells did not exhibit miR34b-modulation of Sox2 expression (Fig 6A), and LNCaP cells had no detectable levels of Sox2 (S4 Fig). Finally, cell proliferation was not significantly affected under the different conditions tested (Panel D in S3 Fig).

When analyzed in human tissues (Fig 6B), Sox2 displayed highly heterogeneous levels of expression especially in PCa tissues ranging from 0% to 70% of positive nuclei (Fig 1C). Conversely, PIN or BPH lesions exhibited high Sox2 immunoreactivity in basal myoepithelial cells (not scored) whereas their epithelial compartments displayed low Sox2 level (≤10%; Sox2 score = 1; Fig 1C). As previously documented [31,36,38], immunoreactivity for Sox2 was anatomically confined to myoepithelial/basal cells in non-neoplastic glands. Conversely, this pattern was progressively lost in PIN and PCa samples. Indeed Sox2 expression in more than 10% of the nuclei (Sox2 scores 2 and 3) was a characteristic of PCa compared to PIN or BPH samples (p = 0.02 by Chi-square test). In line with this result, higher Sox2 scores were more frequently found in PCa tissues of patients who experienced biochemical relapse (p = 0.0006 by Chi-square test; Fig 6D). Indeed the majority of Sox2-negative PCa (59 out of 68, 87%) did not experience biochemical relapse opposite to PCa with Sox2 in more than 10% of the cells (1 out of 6, 0.17%; Fig 6D).

No other clinical variable examined significantly correlated with Sox2 expression.

## Discussion

In this study, we have shown that loss of miR-34b is associated with progression of prostate cancer, and can accurately discriminate between benign hyperplasia and PIN lesions or infiltrating prostatic adenocarcinoma in humans. Mechanistically, this pathway reflects epigenetic silencing and DNA copy number loss of the MIR34B/C locus on chromosome 11, resulting in deregulated expression of the downstream stemness target, Sox2 in PCa. Importantly, deregulated miR-34b signaling appears to selectively segregate with androgen-dependent prostate cells, suggesting a potential role of this pathway in disease relapse and potentially in the transition to castrate-resistant stage.

The miR-34 family of miRNAs has been previously reported to suppress tumorigenesis by different mechanisms, including modulation of cell cycle transitions, EMT, metastasis, or cancer stemness [33]. Consistent with these observations, we have shown here that forced expression of miR-34b potently suppressed the endogenous levels of the stemness factor, Sox2 [33,34], selectively in androgen-dependent cells. Consequently, loss of miR-34b inversely correlated with Sox2 expression in PCa and PIN lesions in humans, while undetectable in normal or hyperplastic epithelium and weakly expressed in myoepithelial cells as also previously described [31,36,38]. In our analysis, high levels of Sox2 correlated with biochemical relapse of PCa, consistent with other data that Sox2 expression segregates with high histologic grade and Gleason score [37]. More work is required to determine how derepression of Sox2 contributes to PCa progression. In previous studies, the expression of stemness markers, including Sox2 has been observed in prostate cancer [39], and linked to disease progression and unfavorable outcome [35]. A previous report [36] documented that Sox2 was repressed by AR in hormone-sensitive PCa. Our data show that Sox2-negative PCa less frequently relapse than patients with high Sox2 immunoreactivity (>10% of positive nuclei). Altogether this evidence suggests that miR-34b/Sox2 axe might be involved in the transition from indolent disease (hormone-sensitive PCa) to more aggressive phases.

However, the existence of a single, defined subset of prostate cancer-initiating cells, or “stem” cells [40], potentially responsible for treatment resistance [41], has not been unequivocally demonstrated [42] and generated conflicting results [43]. Our findings that deregulation of a miR-34b/Sox2 axis occurs early during disease progression, selectively in androgen-dependent cells, suggests that this process may contribute to resistance to androgen ablation therapy, thus heralding an incurable disease stage.

Although the TRAMP model has limitations as a disease surrogate [44], TRAMP mice have been successfully used to identify circulating miRs potentially linked to metastatic disease in humans, including mmu-miR-141, -298, -346 and mmu-miR-375 [45]. Here, the pattern of miRNA expression in PIN or tumor lesion from TRAMP mice was comparable to the profile of invasive, hormone-insensitive DU145 and PC3 cells, and opposite to the androgen-responsive LNCaP cell line. These data validate the use of LNCaP cells as a preclinical model of androgen-dependent prostate cancer [44], whereas androgen-independent cell lines and lesions formed in TRAMP mice more faithfully resemble the more aggressive and poorly differentiated human disease. In this context, a previous study that grouped together LNCaP, DU145 and PC3 cells identified downregulation of miR-130a, -203, -205 as a signature of castrate-resistant and metastatic prostate cancer [46]. Although our results confirmed a lower expression of miR-203 and miR-205 in DU145 and PC3 cells, only miR-205 was significantly decreased in LNCaP cells and human PCa samples, in agreement with previous observations [22,47,48].

In summary, using an integrated discovery platform that included defined cell lines, a genetic mouse model of aggressive disease and clinically annotated human samples, we identified downregulation of miR34b and reciprocal increased levels of Sox2 as a biomarker of progressing prostate cancer while still at an androgen-dependent stage. These findings may offer a straightforward molecular signature to identify patients at risk of aggressive disease, whereas it may be possible to therapeutically manipulate miR-34b levels as a strategy to oppose disease progression [49].

## Supporting Information

S1 Fig TRAMP mice histology and laser-assisted microdissection (LMD) of epithelial tissues. Prostate intraepithelial neoplasia (PIN) or invasive adenocarcinoma of the prostate (PCa) from TRAMP mice (n = 5) was identified by hematoxylin and eosin staining (H&E;) and then isolated by laser-assisted microdissection (LMD) for miRNA profiling. Lesions with neuroendocrine differentiation were therefore excluded from molecular analyses. Scale bar indicates 100μm.
(TIF)

S2 Fig miR-34b-3p levels correctly discriminate between benign and neoplastic prostatic lesions. Receiver operating curves (ROC) analysis was used to assess the accuracy of miR-34b-3p to discriminate between prostatic intraepithelial neoplasia (PIN), or prostatic carcinoma (PCa) and benign prostatic hyperplasia (BPH).
(TIF)

S3 Fig miR-34b expression modulation in prostate cell lines. **(Panel A**) The indicated cell lines were transfected with precursor miR-34b (p-miR-34b-3p), miR-34b inhibitor (a-miR-34b-3p) or with a non-targeting molecule (Ctrl) and analyzed for miR-34b-3p expression by qPCR. Bars, mean±SD; RQ, miRNA relative quantity. **(Panels B, C)** Analysis of miR-34b-3p potential targets. Protein levels of the predicted target c-Myc and Notch1 were analyzed by western blotting **(Panel B)** in the indicated cell lines modulated for miR-34b levels. β-tubulin was a loading control**(Panel C)** Heatmap of miR-34b-3p predicted target genes (c-Myc, Met and Sox2) or of known Notch1 responsive genes (Hes-1 and CDKN1A) in prostate cell lines transfected with precursor, antagonist miR-34b or control molecules as in panel B. Red and blue represent high or low gene expression, respectively. **(Panel D)** Non tumoral (RWPE-1 and BPH-1) or tumoral (LNCaP and DU145) prostate cells were transfected with miR-34b mimic and inhibitor molecules or control and analyzed for cell viability after 72h by direct cell counting. Bars, mean±SEM of three independent experiments. p-miR-34b, precursor-miR-34b; a-miR-34b, antagomiR-34b; Ctrl, mock-transfected control.
(TIF)

S4 Fig Absence of endogenous Sox2 expression in LNCaP cells. A549 (lung cancer) or LN229 (glioblastoma) cell cultures were used as controls for low or high Sox2 expression levels, respectively. β-tubulin was a loading control.
(TIF)

S1 File Raw data_TLDA_PCa Cell lines. (XLSX)

S2 File Raw data_TLDA_TRAMP mouse. (XLSX)

S1 Table miRNA differentially expressed between PCa and PIN lesions of TRAMP prostates. Presence of orthologous murine miRNA in human genome (n = 61) is also reported.
(DOCX)

S2 Table Summary of epigenetic and genomic status of MIR34B/C locus according to miR-34b expression in the indicated prostate tissues or cell lines. (DOCX)
