# Supplementary figures and images for: Deregulation of MiR-34b/Sox2 Predicts Prostate Cancer Progression
Source: PLoS One. 2015 Jun 24;10(6):e0130060. doi: 10.1371/journal.pone.0130060 (PMC4479381; doi:10.1371/journal.pone.0130060)

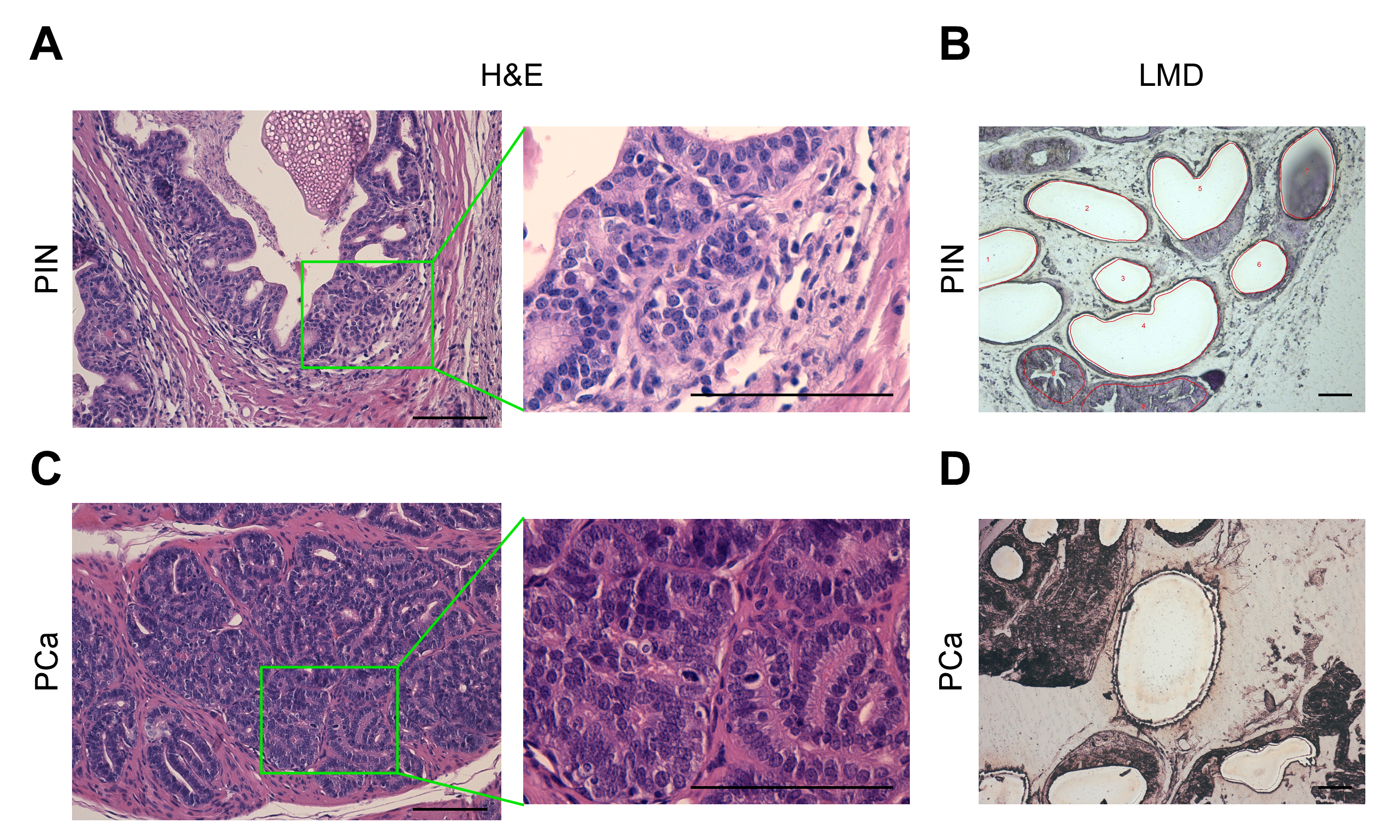

Supplement: S1 Fig — Prostate intraepithelial neoplasia (PIN) or invasive adenocarcinoma of the prostate (PCa) from TRAMP mice (n = 5) was identified by hematoxylin and eosin staining (H&E;) and then isolated by laser-assisted microdissection (LMD) for miRNA profiling. Lesions with neuroendocrine differentiation were therefore excluded from molecular analyses. Scale bar indicates 100μm. (TIF) [file pone.0130060.s001.tif]

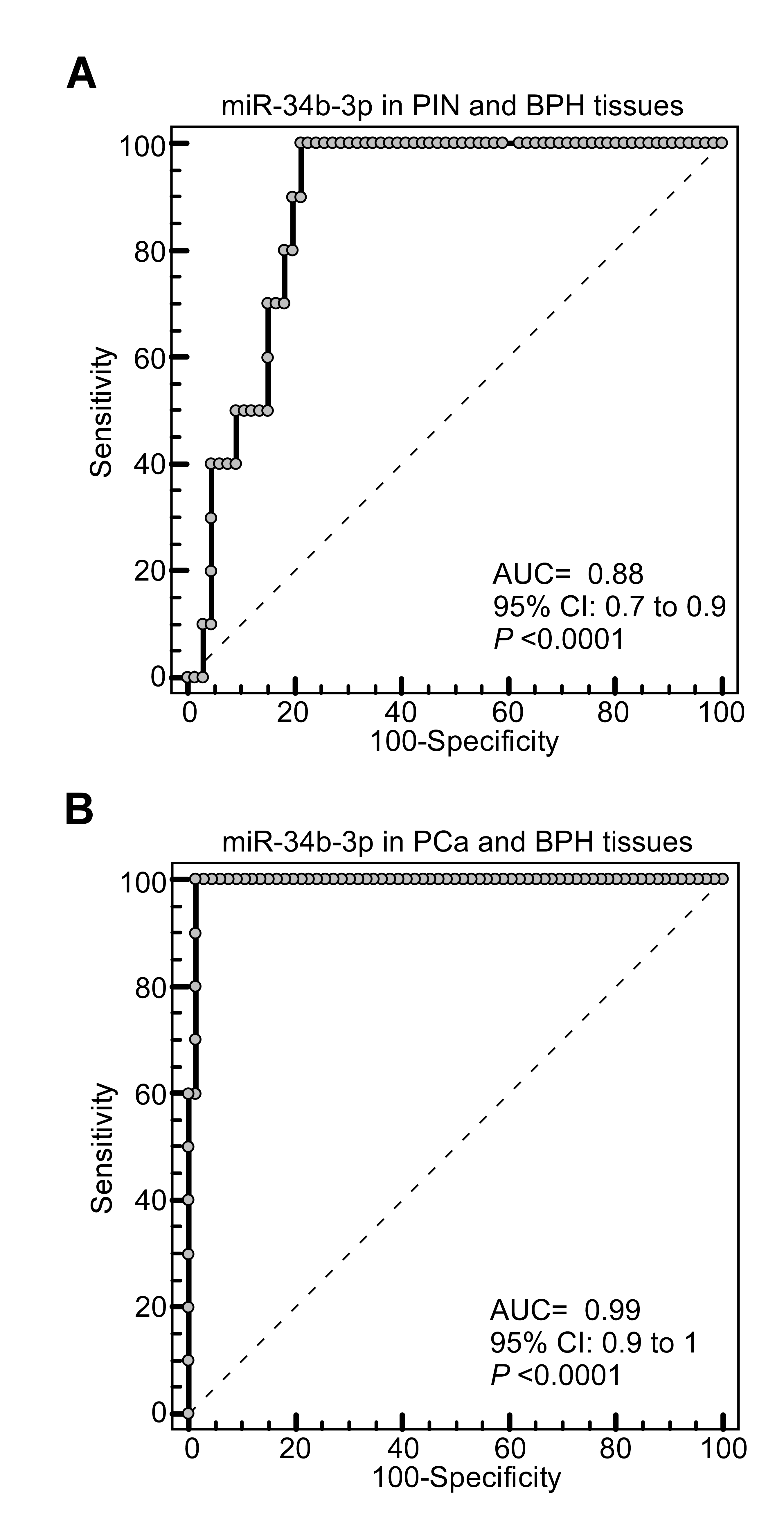

Supplement: S2 Fig — Receiver operating curves (ROC) analysis was used to assess the accuracy of miR-34b-3p to discriminate between prostatic intraepithelial neoplasia (PIN), or prostatic carcinoma (PCa) and benign prostatic hyperplasia (BPH). (TIF) [file pone.0130060.s002.tif]

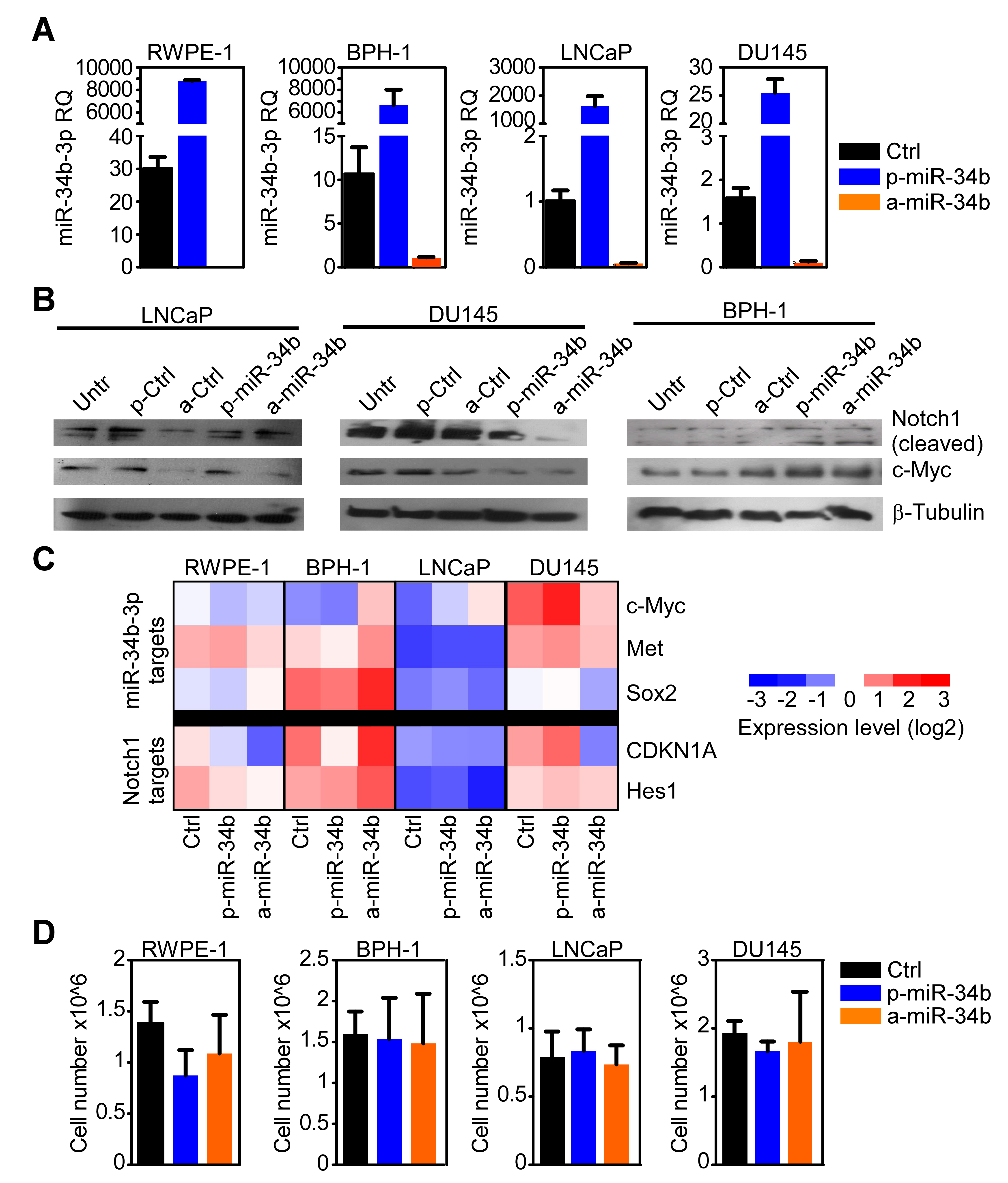

Supplement: S3 Fig — (Panel A) The indicated cell lines were transfected with precursor miR-34b (p-miR-34b-3p), miR-34b inhibitor (a-miR-34b-3p) or with a non-targeting molecule (Ctrl) and analyzed for miR-34b-3p expression by qPCR. Bars, mean±SD; RQ, miRNA relative quantity. (Panels B, C) Analysis of miR-34b-3p potential targets. Protein levels of the predicted target c-Myc and Notch1 were analyzed by western blotting (Panel B) in the indicated cell lines modulated for miR-34b levels. β-tubulin was a loading control(Panel C) Heatmap of miR-34b-3p predicted target genes (c-Myc, Met and Sox2) or of known Notch1 responsive genes (Hes-1 and CDKN1A) in prostate cell lines transfected with precursor, antagonist miR-34b or control molecules as in panel B. Red and blue represent high or low gene expression, respectively. (Panel D) Non tumoral (RWPE-1 and BPH-1) or tumoral (LNCaP and DU145) prostate cells were transfected with miR-34b mimic and inhibitor molecules or control and analyzed for cell viability after 72h by direct cell counting. Bars, mean±SEM of three independent experiments. p-miR-34b, precursor-miR-34b; a-miR-34b, antagomiR-34b; Ctrl, mock-transfected control. (TIF) [file pone.0130060.s003.tif]

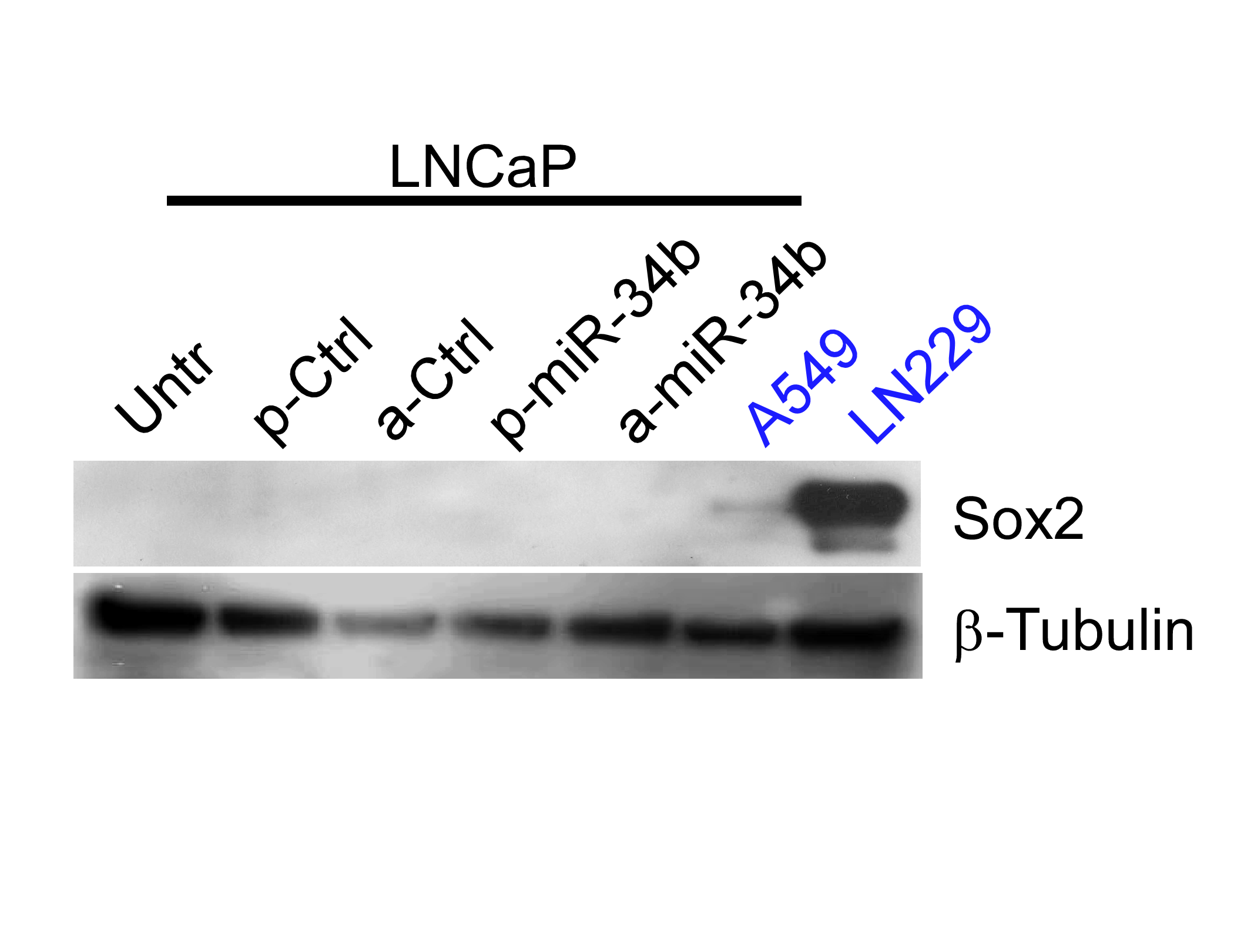

Supplement: S4 Fig — A549 (lung cancer) or LN229 (glioblastoma) cell cultures were used as controls for low or high Sox2 expression levels, respectively. β-tubulin was a loading control. (TIF) [file pone.0130060.s004.tif]
