# Supplementary material for: Deregulation of MiR-34b/Sox2 Predicts Prostate Cancer Progression
Source: PLoS One. 2015 Jun 24;10(6):e0130060. doi: 10.1371/journal.pone.0130060 (PMC4479381; doi:10.1371/journal.pone.0130060)
Supplement: S1 Table — Presence of orthologous murine miRNA in human genome (n = 61) is also reported. (DOCX) [file pone.0130060.s007.docx]

|  | **miRNA name** | **Orthologous in Humans^[[1]](#footnote-1)^** |
| --- | --- | --- |
| **Over-expressed in PCa** | let-7e-3p | Y |
|  | miR-144 | Y |
|  | miR-190b | Y |
|  | miR-206 | Y |
|  | miR-29b-2-3p | Y |
|  | miR-338 | N |
|  | miR-9-3p | Y |
|  | miR-1191 | N |
|  | miR-124 | N |
|  | miR-127 | Y |
|  | miR-129-3p | N |
|  | miR-132 | Y |
|  | miR-134 | N |
|  | miR-136 | Y |
|  | miR-137 | N |
|  | miR-138-3p | N |
|  | miR-138 | Y |
|  | miR-1904 | N |
|  | miR-191-3p | N |
|  | miR-1982.2 | N |
|  | miR-224 | Y |
|  | miR-296-5p | Y |
|  | miR-297a-3p | N |
|  | miR-322 | N |
|  | miR-323-3p | N |
|  | miR-335-3p | Y |
|  | miR-335-5p | Y |
|  | miR-337 | N |
|  | miR-337-5p | N |
|  | miR-338-3p | Y |
|  | miR-344 | N |
|  | miR-34b-3p | Y |
|  | miR-34b-5p | Y |
|  | miR-34c | Y |
|  | miR-362-3p | N |
|  | miR-370 | Y |
|  | miR-374-5p | Y |
|  | miR-376a | N |
|  | miR-376b-3p | N |
|  | miR-376c | N |
|  | miR-380-5p | N |
|  | miR-382 | Y |
|  | miR-384-3p | N |
|  | miR-384-5p | N |
|  | miR-410 | Y |
|  | miR-434-3p | N |
|  | miR-434-5p | N |
|  | miR-449a | Y |
|  | miR-449b | Y |
|  | miR-450b-3p | Y |
|  | miR-466k | N |
|  | miR-485-3p | N |
|  | miR-486 | Y |
|  | miR-487b | Y |
|  | miR-495 | N |
|  | miR-500 | Y |
|  | miR-503 | Y |
|  | miR-539 | Y |
|  | miR-541 | Y |
|  | miR-544 | Y |
|  | miR-592 | Y |
|  | miR-667 | N |
|  | miR-669n | N |
|  | miR-701 | N |
|  | miR-743a | N |
| **Under-expressed in PCa** | miR-143 | Y |
|  | miR-148a-3p | Y |
|  | miR-213 | Y |
|  | miR-214 | Y |
|  | miR-218-2-3p | Y |
|  | miR-22-3p | Y |
|  | miR-27a-3p | Y |
|  | miR-493-3p | N |
|  | miR-875-5p | Y |
|  | miR-105 | Y |
|  | miR-122 | Y |
|  | miR-139-3p | Y |
|  | miR-142-5p | Y |
|  | miR-1905 | N |
|  | miR-1951 | N |
|  | miR-196a-3p | Y |
|  | miR-1981 | N |
|  | miR-199a-5p | Y |
|  | miR-199b | Y |
|  | miR-207 | N |
|  | miR-219 | Y |
|  | miR-23a | Y |
|  | miR-294 | N |
|  | miR-297b-5p | N |
|  | miR-297c | N |
|  | miR-302a | Y |
|  | miR-302d | Y |
|  | miR-30b-3p | Y |
|  | miR-326 | Y |
|  | miR-329 | N |
|  | miR-367 | Y |
|  | miR-377 | Y |
|  | miR-383 | Y |
|  | miR-423-5p | Y |
|  | miR-465a-3p | N |
|  | miR-465a-5p | N |
|  | miR-466a-3p | N |
|  | miR-466e-5p | N |
|  | miR-466j | N |
|  | miR-467c | N |
|  | miR-470 | N |
|  | miR-496 | N |
|  | miR-511 | Y |
|  | miR-546 | N |
|  | miR-574-3p | Y |
|  | miR-669a | N |
|  | miR-669c | N |
|  | miR-692 | N |
|  | miR-694 | N |
|  | miR-698 | N |
|  | miR-702 | N |
|  | miR-743b-3p | N |
|  | miR-761 | Y |
|  | miR-763 | N |
|  | miR-871 | N |
|  | miR-98 | Y |

1. According to Ensembl Genome Browser web application (<http://www.ensembl.org/index.html>). Y, presence of orthologous miRNA; N, absence of orthologous miRNA. [↑](#footnote-ref-1)
