# Supplementary material for: Deregulation of MiR-34b/Sox2 Predicts Prostate Cancer Progression
Source: PLoS One. 2015 Jun 24;10(6):e0130060. doi: 10.1371/journal.pone.0130060 (PMC4479381; doi:10.1371/journal.pone.0130060)
Supplement: S2 Table — (DOCX) [file pone.0130060.s008.docx]

| **Sample category** | | **miR-34b levels** | **Methylated MIR34b/c CpG (n)** | **CNV#1 loss (n)** | **CNV#2 loss (n)** | **CNV#3 loss (n)** |
| --- | --- | --- | --- | --- | --- | --- |
| **Tissues** | PCa | Low: n=7 | 5 | 4 | - | - |
|  |  | High: n=3 | 1 | 2 | - | - |
|  | BPH | Low: n=0 | - | - | - | - |
|  |  | High: n=10 | 3 | 1 | - | - |
| **Cell lines** | RWPE-1 | High | No | No | No | No |
|  | BPH-1 | Low | Yes | Yes | Yes | No |
|  | LNCaP | Low | No | Yes | Yes | No |
|  | DU145 | High | No | No | No | No |
